# Can theory of mind of healthy older adults living in a nursing home be improved? A randomized controlled trial

**DOI:** 10.1007/s40520-021-01811-4

**Published:** 2021-03-08

**Authors:** Elena Cavallini, Irene Ceccato, Silvana Bertoglio, Andrea Francescani, Federico Vigato, Aladar Bruno Ianes, Serena Lecce

**Affiliations:** 1 Department of Brain and Behavioral Sciences, University of Pavia, Pizza Botta 6, 27100 Pavia, Italy; 2 Department of Neuroscience, Imaging and Clinical Sciences, University “G. D’ Annunzio” of Chieti-Pescara, Via dei Vestini 31, 66100 Chieti, Italy; 3 Nuova Assistenza, Via Rudinì 3, 20142 Milan, Italy; 4 Fornaroli Hospital, Via al Donatore di Sangue 50, 20013 Magenta, MI Italy; 5 Korian, Viale Cassala 16, 20143 Milan, Italy

**Keywords:** Intervention, Training, Mentalizing, Socio-cognitive abilities, Transfer effects

## Abstract

**Background:**

Research in nursing homes mainly focused on interventions for residents affected by cognitive decline. Few studies have considered healthy older adults living in nursing homes, and this research targeted cognitive functioning.

**Aims:**

To evaluate whether socio-cognitive abilities can be improved by means of a theory of mind (ToM) training conducted by nursing home’s operators.

**Methods:**

**Results:**

Results revealed that older adults benefitted from the ToM intervention in both practiced and non-practiced tasks, while the control group showed no change from pre- to post-test evaluation. Analyses on errors scores indicated that the ToM intervention led to a reduction of both excessive mentalizing and absence of mental states inference.

**Discussion:**

The conversation-based ToM intervention proved to be effective in improving socio-cognitive skills in cognitively healthy nursing home residents. Notably, older adults were able to transfer the skills acquired during the training to new material.

**Conclusions:**

Promoting healthy resident’s ToM ability could positively impact on their social cognition, consequently increasing their quality of life. Our findings showed that the intervention can be feasibly managed by health care assistants within the residential context.

**Supplementary Information:**

The online version contains supplementary material available at 10.1007/s40520-021-01811-4.

## Introduction

As the number of older adults in the world is increasing, there will be a commensurate increase in the demand for nursing homes (NH). Older adults living in NH represent a heterogeneous population. Notwithstanding a high number of older adults is affected by some type of neurodegenerative and cognitive disease, other NH residents present a physiological cognitive decline. Both the clinical and non-clinical populations of older people resident in NH need to be involved in stimulating activities, as they tend to be inactive and engage in sedentary activities for most of the day [1].

Research has mainly focused on interventions for NH residents affected by cognitive decline, such as dementia [2]. Only a few studies have taken into account healthy older adults living in residential care homes [3, 4]. This research, focusing on fostering cognitive functioning, revealed that interventions based on cognitive activities are capable of promoting resident’s performance.

The present study takes as its premise the acknowledgment that resident’s social involvement is crucial for their health, as it impacts on people’s quality of life [5], and is associated with life meaning, life satisfaction, and psychological well-being [6]. Some studies have reported that social engagement puts residents at a lower risk of negative physical and mental health outcomes, including depression, loneliness, and death [7, 8]. In addition, close relationships between staff and residents and between residents and peers influence perceptions of the quality of care and the experience of a positive feeling of personal growth [9, 10]. Hence, potentiating the skills underlying good relationships is crucial for residents’ social functioning.

One core dimension of these skills is Theory of Mind (ToM), the ability to recognize others’ mental states (thoughts and emotions) and to predict others’ behavior [11]. Findings from typical and atypical development showed that ToM is crucial to successfully navigate the social world [12]. ToM helps people to build and maintain positive social relationships, as it permits the understanding of complex social scenarios and of other’s needs; it reduces the risk of misunderstanding while increasing the level of social competence [13].

Older people have difficulties in inferring mental states implicated in complex social scenarios, such as faux pas, misunderstanding, and deception [14–16]. Notably, recent research in aging has shown that the decline in social functioning [17, 18] and social intelligence [19], is, at least partly, attributable to a reduction in socio-cognitive skills. The association between ToM and social adjustment has also been identified among older people living in NH: higher social cognitive skills predicted nurse’s ratings of resident’s social functioning [20]. These findings suggest that ToM is an essential prerequisite for good interpersonal functioning in aging, and that its decline is potentially critical for older people’s social adjustment which, in turn, impacts on cognitive and physical functioning [21, 22].

Recent studies on ToM training interventions in aging have shown that community-dwelling older adults can improve their ToM performance [23–25]. Results of this body of research also demonstrated that verbal knowledge predicts improvements in practiced tasks and baseline performance along with executive functioning and age predict gains on the transfer task, highlighting that older adults need more resources to generalize their behavior to new tasks [26]. Despite these interesting results, no research on ToM training has been conducted in the NH population.

The aim of the present study was to evaluate whether the ToM training was effective in fostering NH healthy residents’ ToM ability. In this study, we administered a conversation-based ToM intervention that has been proved to successfully improve older adults’ ToM. This intervention is based on group conversations about people’s thoughts and emotions guided by the trainer through: (a) frequent use of mental-state terms [27, 28]; (b) focusing on the dynamic nature of the mind; (c) reflections on daily life situations similar to those presented in the exercises. Special attention was given to the dynamic nature of thoughts and emotions, stimulating participants to find solutions to resolve complex social situations, such as misunderstandings and quarrels. Reflection on personal experiences of real-life situations makes the activities more meaningful and helps participants realize that those skills improved during the training can be transferred to daily life [29]. More details on activities and materials can be found in previous studies [23].

In the present study, we adapted the materials of Cavallini and colleagues to our sample of NH residents by reducing the task’s complexity and length, as the age range of our sample was older than that in previous studies (past studies’ average age ranging from 64.41 to 75.76; current sample: *M*_age_ = 83.77). To measure the training effects, we used the Strange Stories task (SST) as the practiced task [30] and the Movie for the Assessment of Social Cognition [31, 32] as the transfer task able to capture mentalizing errors.

Given that previous results proved the efficacy of the ToM training in community dwelling older adults, even in the older group [24], and the usefulness of cognitive interventions in the NH population [3, 4], we predicted that our conversation-based ToM intervention would generate gains for the trained group in the practiced as well as the non-practiced task. Since we adapted the training materials according to the grade of older adult’s cognitive resources, we expected that all participants would benefit from our intervention. Regarding the error type, given that our training increases participant’s awareness of and ability to reason about mental states, we expected a reduction of no-ToM and iper-ToM errors, as they represent the more extreme failures in mental state reasoning.

## Materials and methods

### Participants

A total of 31 healthy older adults participated in the study (Table 1). Residents were recruited from 5 NH (with approximately 80 residents, about 10 of them are cognitively healthy) located in the North of Italy using the following inclusion criteria: (a) no psychiatric or neurological diseases; (b) no cognitive impairment, as indicated by adjusted scores on the MMSE higher than 24 [33]; (c) no depressive symptomatology, as indicated by scores lower than 23 on the CES-D [34]. No tangible incentives were given to participate. Only motivated and interested residents were included in the study. Participants were randomly assigned to one of two conditions: a conversation-based ToM training group, and an active control training group. Older adults within the same NH were assigned to different conditions. Preliminary separate one-way analyses of variance were performed to establish the equivalence of the two groups before the training (i.e., pre-test), and, for CES-D, at the end of the training (i.e., post-test). Results indicated that the two groups were equivalent (Table 1).

**Table 1:** Demographic and background participant’s characteristics as a function of the intervention group

	ToM training *n* = 16			Active Control training *n* = 15			Group differences	
	*M*	*SD*	*range*	*M*	*SD*	*range*	*F* (1, 29)	$\eta_{{\text{p}}}^{2}$
Age	82.69	8.81	66–95	84.93	6.93	68–95	0.62	0.02
% Female	56.3			60.0			0.05^a^	0.04^b^
Years of education	8.25	4.51	3–18	8.13	3.83	4–18	0.01	0.00
Vocabulary	30.50	12.36	14–46	25.93	13.86	5–47	0.94	0.03
MMSE	28.22	1.25	26–30	28.42	1.15	26.8–30	0.20	0.01
CES-D								
Pre-test	10.81	4.82	3–21	12.93	6.47	1–22	1.08	0.04
Post-test	8.06	4.09	3–16	11.33	5.65	1–18	3.44	0.11

*M* mean, *SD* standard deviation

^a^Chi-square is reported, with *df* = 1

^b^As a measure of effect size Cramer’s V is reported

*p < 0.05, **p < 0.01, ***p < 0.001

### Materials

Background information was obtained with a demographic questionnaire. As control variables, we evaluated general cognitive functioning (MMSE), crystallized intelligence (Vocabulary subtest taken from the PMA), and depressive symptomatology (CES-D). Please refer to Online Resource for further details.

To measure ToM, we selected two tasks previously used with older adults, to examine both practice and transfer effects of the training. The Strange Stories task (SST) was the task trained in the conversation-based ToM intervention, while the MASC was not presented during the training activities, thus representing the non-practiced task. The SST requested to read and interpret short written social scenarios. The MASC is a video-based, ecologically valid task. It requests to watch social interactions and answers to questions about character’s mental states in a multiple-choice format. Notably, this task also allows investigating the type of error made: among the four response alternatives given, the three wrong answers reflect three types of errors. Hence, four scores were computed: the percentages of accurate answers (MASC accuracy), as an index of ToM ability, and three error scores. Iper-ToM indicated an excessive attribution of mental states when not necessary; ipo-ToM reflected a correct but insufficient attribution of mental states that prevented the full understanding of the social situation; and no-ToM indicated a complete lack of mental states attribution (see Table S1 in Online Resource for details).

### Procedure

All participants were pre-tested on control variables and ToM tasks before the training. Both interventions consisted of four 1 h collective sessions at one-week intervals, carried out in a quiet room within the NH. At the end of the training program, participants were post-tested on ToM tasks. Crucially, the training activities were conducted by NH’s qualified (psychologists and educators) staff, who were trained before the interventions (see Online Resource for details). The researcher monitored the fulfilment of the intervention.

The conversation-based ToM training program was based on that of Lecce and colleagues [25]. Detailed activities and procedures are presented in the Online Resource. Participants in the active control group took part in cognitive activities based on newspaper reading, crossword puzzles, and text writing.

### Data analyses plan

To evaluate group-related differences at baselines, two one-way ANOVAs were computed on practiced and non-practiced tasks as a function of the group (ToM vs. control). Subsequently, we evaluated training benefits. For the practiced task, due to the difference between the two groups in the baseline performance, we carried out an ANCOVA on the post-training score, covarying for pre-test score. For the non-practiced task, we performed a series of two (Group: ToM vs. control) by two (Time: pre-test vs. post-test) mixed design ANOVAs on MASC accuracy and error scores. Assumptions of normality and homogeneity of variance were generally met, with few exceptions. However, the equal size of the two groups makes these assumptions less stringent [35]. Finally, to test whether individual differences in background variables modulated the training benefits, hierarchical linear regression analyses were conducted for the conversation-based ToM intervention. For each ToM task, the baseline performance (pre-test) was entered at the first step, and age and MMSE scores at the second step. We checked tolerance and VIF values and found no evidence of collinearity within our data.

Sensitivity analyses were conducted after data collection to compute the minimum effect size that can be detected given alpha, power, and sample size [36]. We used G*Power and set sample size of 31 participants (16 for the regression analyses), an alpha level of 0.05, and a minimum power of 0.80. For the ANCOVA, results revealed we have enough power to detect $\eta_{{\text{p}}}^{2}$  ≥ 0.21. For the mixed-design ANOVA, we have enough power to detect $\eta_{{\text{p}}}^{2}$ ≥ 0.06 for the within-subject main effect, and $\eta_{{\text{p}}}^{2}$ ≥ 0.05 for the interaction. For the regression, we have enough power to detect a *R*^2^ ≥ 0.49 for the whole model, and a Δ*R*^2^ ≥ 0.44 considering two additional predictors.

The dataset is freely available in OSF repository: https://osf.io/5rxvu/?view_only=ab8c36783e1f46d589a98e5e087e23f0.

## Results

### Preliminary analyses

Regarding the practiced task (SST), we found significant differences between the two groups at the pre-test, *p* = 0.014, indicating that the ToM group performed better compared to the control group. For the non-practiced task (MASC), the two groups did not differ in accuracy, *p* = 0.763, nor in error score, *ps* ≥ 0.331 (Table 2).

**Table 2:** Descriptives and group differences between ToM training and active control training groups in practiced and non-practiced ToM tasks

	ToM training group				Control group				Group difference at pre-test			Group differences at post-test^a^	
	Pre-test		Post-test		Pre-test		Post-test						
	*M*	*SD*	*M*	*SD*	*M*	*SD*	*M*	*SD*	*F*	$\eta_{{\text{p}}}^{2}$	*F*		$\eta_{{\text{p}}}^{2}$
SST	53.12	18.48	78.65	19.24	35.00	19.97	33.33	27.64	6.89*	0.19		17.95***	0.39
MASC accuracy	50.54	17.74	76.14	24.48	48.70	15.86	48.49	30.76	0.09	0.00	Time: Time*group:	11.85** 12.25**	0.29 0.30
iper-ToM	21.74	10.65	8.52	12.07	22.32	11.49	17.88	13.69	0.02	0.00	Time: Time*group:	22.62*** 5.59*	00.44 0.16
ipo-ToM	13.86	7.65	8.81	9.01	16.81	8.98	16.36	10.42	0.97	0.03	Time: Time*group:	2.27 1.59	0.07 0.05
no-ToM	13.86	7.97	6.25	9.52	12.17	4.98	17.27	13.76	0.49	0.02	Time: Time*group:	0.27 6.99*	0.01 0.19

^a^For the SST score, we ran an ANCOVA controlling for baseline performance. For MASC scores, we ran 2 × 2 repeated measure ANOVAs, with Time (pre- vs post-test) as within-subject factor and Group (ToM vs. Control) as between-subject factor. *p < 0.05, **p < 0.01, ***p < 0.001

Interestingly, the repeated measures ANOVA on error type (three levels: iper-ToM, ipo-ToM, no-ToM), taking into consideration all participants across the two groups, reported significant differences among error types, *F*(2, 29) = 6.75, *p* = 0.004, $\eta_{{\text{p}}}^{2}$ = 0.32. Pairwise comparisons indicated that iper-ToM errors were significantly more frequent than ipo-ToM errors, *p* = 0.016, and no-ToM errors, *p* = 0.002. Ipo­ToM and no-ToM errors were equally frequent, *p* = 0.551.

### Interventions effects on the ToM tasks

#### Practiced task

**Fig. 1:**
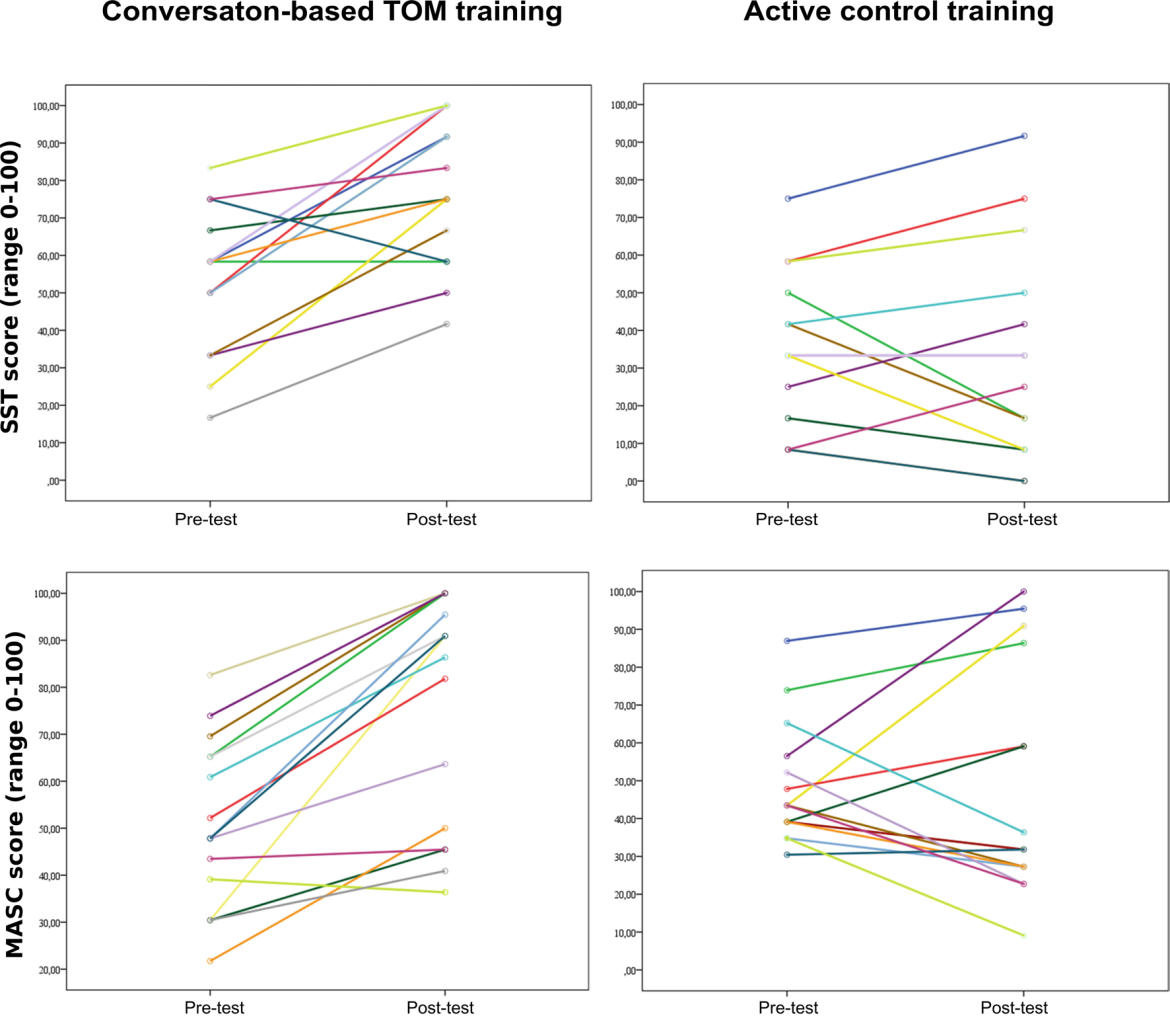
Spaghetti plot depicting individual trajectories of change from pre-test to post-test in the practiced task (above), and in the non-practiced task (below), separated for the two intervention groups. Each line represents a single participant

Results revealed that the conversation-based ToM training group outperformed the control group, *p* < 0.001, even when controlling for the baseline performance. Almost all participants in the ToM training increased their performances at post-test (Fig. 1, upper, left). Alternatively, the control group showed a not consistent pattern of change (Fig. 1, upper, right), with the majority of individuals showing maintenance of their performance.

#### Non-practiced task

Results showed an improvement from pre- to post-test, *p* = 0.002. More interestingly, we found a significant interaction effect, *p* = 0.002. Pairwise comparisons indicated that only the conversation-based ToM training improved from pre- to post-test, *F*(1, 29) = 24.91, *p* < 0.001, $\eta_{{\text{p}}}^{2}$ = 0.46, while no differences emerged in the control group, *F*(1, 29) = 0.00, *p* = 0.969, $\eta_{{\text{p}}}^{2}$ = 0.00. Figure 1 (lower) shows a nuanced pattern, with the ToM individuals showing a positive change, and the control group showing a less clear pattern of individual trajectories.

Analyses on the types of errors revealed a significant interaction effect, *p* = 0.025 for iper-ToM errors, showing a decrease of these errors in the conversation-based ToM group. Pairwise comparisons reported a reduction of iper-ToM error in the ToM training group, *F*(1, 29) = 26.19, *p* < 0.001, $\eta_{{\text{p}}}^{2}$ = 0.47, and no significant changes in the control group, *F*(1, 29) = 2.77, *p* = 0.107, $\eta_{{\text{p}}}^{2}$ = 0.09. For ipo-ToM errors, neither the main effect of Time nor the Time by Group interaction was significant, *p* ≥ 0.143. The pairwise analysis showed that the conversation-based ToM group showed a reduction in ipo-ToM scores that approached statistical significance, *F*(1, 29) = 3.96, *p* = 0.056, $\eta_{{\text{p}}}^{2}$ = 0.12. For no-ToM errors, results showed a significant Time by Group interaction, *p* = 0.013, with the conversation-based ToM group, but not the Control group, reporting a significant decrement in no-ToM errors, *F*(1, 29) = 5.18, *p* = 0.030, $\eta_{{\text{p}}}^{2}$ = 0.15.

### Predictors of ToM performances at post-test

Lastly, we performed regression analyses to examine which variable accounted for individual differences in ToM performances at the end of the conversation-based ToM intervention.

Considering the practiced task, regression analyses reported that the regression model did not show adequate fit to the data (*p* ≥ 0.064). Therefore, none of the entered predictors accounted for a significant amount of variance in post-test score (pre-test scores, *β* = 0.39, *t*(15) = 1.69, *p* = 0.117; age, *β* = − 0.43, *t*(15) = − 1.82, *p* = 0.086; MMSE, *β* = − 0.16, *t*(15) = − 0.699, *p* = 0.498. Considering the non-practiced task, findings indicated that pre-test scores (*β* = 0.74, *t*(15) = 4.177, *p* = 0.001) significantly predicted performance at post-test, *R*^2^ = 0.55, *F*(1, 14) = 17.44, *p* = 0.001. The addition of age (*β* = 0.24, *t*(15) = 1.380, *p* = 0.193) and MMSE in the second step did not significantly improve explained variance, Δ*R*^2^ = 0.14, *F*(2, 12) = 2.81, *p* = 0.100, even if MMSE resulted as a significant predictor (*β* = 0.35, *t*(15) = 2.178, *p* = 0.049).

## Discussion and conclusions

The present study was designed to test the effectiveness of an intervention promoting socio-cognitive abilities in healthy older adults living in a NH, administered via conversation-based ToM training. The ToM training was compared with an active control training, in which participants did cognitive exercises with no specific focus on mental states reasoning. We found that older adults benefitted from the ToM intervention in both practiced and non-practiced tasks, while participants in the control group showed no change from pre- to post-test evaluation.

To test training benefits in the practiced tasks we used an advanced ToM measure, the SST. Notwithstanding the fact that in this task the two groups differed at baseline, with the control group reporting lower performance than the conversation-based ToM group, participants in the ToM training group improved more than the others. This result is in line with previous papers in which Lecce and colleagues found that the ToM training led to improvements in practiced tasks [37]. Although this effect is not surprising, in the present study the sample was older than those considered before, demonstrating that ToM performance can also be enhanced in old age [24].

The most crucial result pertains to transfer effects. Firstly, it suggests that older people living in a NH can improve their ToM abilities in such a way that they are able to transfer the skills acquired during the training to new and different material. The non-practiced task used [31] differs from the practiced task in a range of features, especially in the dynamicity of the stimuli and its similarity with real-life situations. An improvement in this task suggests that older people may be able to transfer their learned ToM skills to daily social exchanges. Since social exchanges are frequent in NH, as residents have to interact with different people, such as roommates, medical staff, etc., this finding could have strong implications for residents’ life [38].

Secondly, the MASC allowed us to execute a more specific investigation into how the use of ToM skills changed. Analyses on error scores indicated that the improvement in ToM accuracy was due to a reduction of iper-ToM and no-ToM errors. Older adults became more capable at avoiding an excessive attribution of mental states when not appropriate, and better at recognizing mental states in understanding social situations. The reduction of iper-ToM errors warrants some attention, as it is the most frequent type of error. Iper-ToM errors could lead to a bias in attributing negative intentions to others, and thus cause social hostilities and conflicts [39], as reported in clinic populations such as (paranoid) schizophrenia, autism spectrum disorders, borderline personality disorder, and social anxiety [40, 41]. Furthermore, the reduction of no-ToM errors reflects an improvement in the ability to detect mental states. In other words, the conversation-based ToM training effectively reduced people’s overlooking of mental states, which are crucial to the understanding of social situations. This is a particularly striking result, as it implies that after the ToM intervention older adults became more adept at interpreting social situations, taking the underlying mental states into account, rather than focusing solely on physical (and irrelevant) information.

We believe that the increase in ToM performance derives from two crucial aspects of the intervention: the conversational approach and the dynamic nature of the training. Regarding the conversational approach, theoretical [42, 43] and empirical findings [44, 45] support the view that conversations help individuals improve their awareness that others have different points of view on the same situation, and make them competent in using their training experience to reflect on the mind. Considering the dynamic nature of the training, our intervention focused on making participants realize that mental states are not static but can change over time. Older adults had the opportunity to understand that mental activity is flexible and can be modified depending on social input.

Finally, we ran regressions analyses to investigate who our conversation-based ToM intervention was best suited for. Outcomes revealed a different pattern of results for both the practiced and non-practiced tasks. For the practiced task, the post-test performance was not predicted by any considered variables, revealing that all participants improved, irrespective of their initial ToM level, MMSE, and age. This suggests that the adaptation of the training activities was successful. For the non-practiced task, the initial ToM level was related to the final performance, indicating that people starting with a higher baseline performance tended to reach a higher post-test level. We also found that the MMSE marginally predicted performance at the post-test. This is in line with previous studies highlighting that the generalization of the trained skills is cognitively demanding [26, 46]. Nevertheless, age was not a predictor of improvement in the non-practiced task, either, suggesting that our ToM intervention is suitable for an old population within a wide age range, when adapted appropriately. However, because of the limited sample size our analyses should be considered with cautions. Since we had enough power to detect only large effects, it may be the small effects of age and cognitive functioning existed, but we failed to find them.

The current study has some limitations that should be considered in future research. First, given the small number of participants, due to the limited number of cognitively healthy residents living in each NH, present findings should be considered as preliminary. Second, given the involvement in the project of the NH staff, also in the assessment phases, we could not run a double-blind study. Replication studies are needed to confirm the ToM training efficacy and to elaborate on individual differences in benefits. Second, we did not measure participants' social functioning. In the future, it may be useful to use a specific measure to better investigate the association between ToM, social relationships and social functioning. In addition, the CES-D at the post-test showed marginally significant differences between the two groups with a medium-to-large effect size. Future studies should take into account potential benefits of ToM training on older adults’ affective states. Notwithstanding these caveats, current findings significantly extend previous research demonstrating ToM training feasibility in NH. Moreover, since close relationships between residents and staff are believed to be essential for understanding resident care preferences and values in order to provide person-centered care [47], and given the association between ToM ability and social relationships in healthy older adults [18, 37, 48], improving ToM ability may support the development of social relationships in resident’s everyday life, with positive consequences on physical and mental health. Finally, we believe that one of the strengths of the conversation-based ToM intervention presented is that it was conducted and managed by the NH staff. We tested the transportability of an effective socio-cognitive intervention within the NH context, subject to slight adaptations in materials and times. Moreover, this intervention can be smoothly implemented in regular activities within the NH, without the need of external professionals.

## Supplementary Information

Below is the link to the electronic supplementary material.

Supplementary file1 (DOCX 32 KB)

## Data Availability

The dataset analyzed in the current study is freely available on the OSF repository, https://osf.io/5rxvu/?view_only=ab8c36783e1f46d589a98e5e087e23f0
