# Supplementary material for: Can theory of mind of healthy older adults living in a nursing home be improved? A randomized controlled trial
Source: Aging Clin Exp Res. 2021 Mar 8;33(11):3029–37. doi: 10.1007/s40520-021-01811-4 (PMC8595145; doi:10.1007/s40520-021-01811-4)
Supplement: Supplementary file 1 — Supplementary file1 (DOCX 32 KB) [file 40520_2021_1811_MOESM1_ESM.docx]

**Can Theory of Mind of healthy older adults living in a nursing home be improved? A randomized controlled trial**

Cavallini, E., Ceccato, I., Bertoglio, S., Francescani, A., Vigato, F., Ianes, A. B., and Lecce, S.

Corresponding author: Elena Cavallini, Department of Brain and Behavioral Sciences, University of Pavia, e-mail: [ecava@unipv.it](mailto:ecava@unipv.it)

**Supplementary Information**

**Table S1.** *Constructs investigated and details on the tasks used.*

| *Construct* | *Task (references)* | *Characteristics* | *Scores* |
| --- | --- | --- | --- |
| Background information | Questionnaire | Items concerning age and years of educations. | - |
| Crystallized intelligence | Vocabulary subset from the PMA  Primary Mental Abilities test (Thurstone & Thurstone, 1963) | Subjects were asked to identify the correct synonymous among five options of 50 target words within 8 minutes. | 0-50 |
| General cognitive functioning | MMSE  Mini Mental State Examination  (Folstein et al., 1975; Magni et al., 1996) | The MMSE is a brief screening tool commonly used in nursing homes as it allows to measure cognitive functioning and to detect cognitive deficits. The items investigate attention/orientation, memory, language and visuospatial abilities. | 0-30 |
| Depressive symptomatology | CES-D Center for Epidemiological Studies Depression Scale  (Fava, 1983; Radloff, 1977) | The CES­D is a self-administered questionnaire consisting of 20 items asking to rate how often over the past week the subject experienced symptoms associated with depression, such as restless sleep, poor appetite, and feeling lonely. Higher scores reflect higher level of depressive symptoms. | 0-60 |
| ToM –  practiced task | Strange stories (Happé et al., 1998; White et al., 2009) | Written stories presenting complex social situations which require ToM ability to be accurately understood. We selected 6 stories from the original set, presenting three social scenarios: double bluff, sarcasm, and misunderstanding. After having read each story, subjects were asked to answer the test question: *Why did the main character say this?* Participants had no time limit to answer.  Stories were available for inspection until a response was given, to reduce memory demands.  According to the scoring procedure participants’ responses were rated using a three-point scale: 0 for incorrect answers, 1 for partially correct answers, and 2 for full and explicit answers. Scores were then transformed into percentages of accuracy.  At post-test we administered a parallel version of the task, with six different stories. | 0-100 |
| ToM –  non-practiced task | MASC  Movie for the Assessment of Social Cognition  (Dziobek et al., 2006; Fossati et al., 2018) | The MASC is a visual-dynamic task in which a 15-minutes video clip is sequentially stopped by questions about the characters’ feelings and thoughts. The plot consists of four characters who are meeting for dinner, with both friendships and romantic issues presented. This task approximates real life situations by depicting familiar topics, integrating different input channels (visual and auditory), and requesting online reasoning. Furthermore, it allows to investigate the typology of error made: for each question, among the four alternatives given, the three wrong answers reflect three possible errors  Four scores were computed: the percentages of accuracy (MASC accuracy), as index of ToM ability, and three error scores (MASC iper-ToM, ipo-ToM, no-ToM), by transforming raw frequencies into percentages.  We divided the original video in two parts, and administered the first part at pre-test (23 items) and the second part at post-test (22 items). | 0-100 |

**Details on the conversation-based ToM training**

A researcher with extensive experience in ToM training intervention for older people conducted 4 operator-training sessions. In these sessions the researcher explained the aim and the structure of the intervention and presented the training’s exercises to familiarize operators with them. The operators received also written guidelines and scripts to conduct group discussions. Specifically, in these guidelines were presented: (1) the exercises of each training session, with the expected time allocation; (2) the strategies to implement these exercises; (3) the input that operators were supposed to give to participants during group discussions. Operators were invited to ask as many questions as they needed, and they were encouraged to discuss any doubts about the content of the training and to suggest exercises’ adaptations to better fit with residents’ daily experiences. We scheduled the first two operator-training sessions the month before the beginning of the training program. The other two operator-training sessions were run during the implementation phase. This allowed the experimenter to monitor the fulfilment of the intervention. Specifically, we scheduled the third operator-training session between the first and the second sessions and the fourth operator-training session between the third and the fourth residents-training session.

**Table S2.** *Conversation-based ToM training activities.*

| The activities increased in the level of complexity lesson after lesson and made use of a range of modalities and materials (visual and written stimuli).  Each exercise firstly had to be carried out individually, then, when all participants had finished their work, they were involved in group conversations lead by the trainer.  During the group conversations, the trainer:  - provided explanations and feedbacks to participants;  - encouraged participants to elicit a complete and explicit understanding of the mental states underlying characters’ behavior;  - made extensive use of mental state verbs (e.g., to think, know, feel), within sentential complement construction;  - promoted the participation of each participant;  - stimulated the awareness of the dynamic nature of mental states, by asking participants to find possible way to resolve the conflicts/difficulties in the social situations presented;  - linked training exercises to daily life experiences, by asking participants to imagine personal situations similar to those presented in the exercises and to reflect on how they did or could manage them.  Five types of exercises were used: | |
| --- | --- |
| *Stories* | short texts similar to those of the Strange Stories task, followed by questions on thoughts and feelings of the protagonists. |
| *Visual*  *perspective taking* | ambiguous visual stimuli that can elicit different interpretations from different individuals. |
| *Mental verbs* | sentences in which participants had to find a synonym for a given mental state. |
| *Pictures for emotions* | drawings depicting familiar scenes, in which the trainer asked participants to identify characters’ emotions based on visual cues. |
| *Conceptual perspective taking* | stories presenting situations extremely similar to daily life, and requesting participants to switch perspectives from that of one protagonist to the other |

**References**

Dziobek, I., Fleck, S., Kalbe, E., Rogers, K., Hassenstab, J., Brand, M., Kessler, J., Woike, J. K., Wolf, O. T., & Convit, A. (2006). Introducing MASC: A Movie for the Assessment of Social Cognition. *Journal of Autism and Developmental Disorders*, *36*(5), 623–636. https://doi.org/10.1007/s10803-006-0107-0

Fava, G. A. (1983). Assessing depressive symptoms across cultures: Italian validation of the CES-D self-rating scale. *Journal of Clinical Psychology*, *39*(2), 249–251. https://doi.org/10.1002/1097-4679(198303)39:2<249::AID-JCLP2270390218>3.0.CO;2-Y

Folstein, M. F., Folstein, S. E., & McHugh, P. R. (1975). “Mini-mental state”: A practical method for grading the cognitive state of patients for the clinician. *Journal of Psychiatric Research*, *12*(3), 189–198. https://doi.org/10.1016/0022-3956(75)90026-6

Fossati, A., Borroni, S., Dziobek, I., Fonagy, P., & Somma, A. (2018). Thinking about assessment: Further evidence of the validity of the Movie for the Assessment of Social Cognition as a measure of mentalistic abilities. *Psychoanalytic Psychology*, *35*(1), 127–141. https://doi.org/10.1037/pap0000130

Happé, F. G. E., Winner, E., & Brownell, H. (1998). The getting of wisdom: Theory of mind in old age. *Developmental Psychology*, *34*(2), 358–362. https://doi.org/10.1037/0012-1649.34.2.358

Magni, E., Binetti, G., Bianchetti, A., Rozzini, R., & Trabucchi, M. (1996). Mini-Mental State Examination: A normative study in Italian elderly population. *European Journal of Neurology*, *3*(3), 198–202. https://doi.org/10.1111/j.1468-1331.1996.tb00423.x

Radloff, L. S. (1977). The CES-D Scale: A self-report depression scale for research in the general population. *Applied Psychological Measurement*, *1*(3), 385–401. https://doi.org/10.1177/014662167700100306

Thurstone, T. G., & Thurstone, L. L. (1963). *Primary mental ability*. Science Research Associates.

White, S., Hill, E., Happé, F., & Frith, U. (2009). Revisiting the Strange Stories: Revealing mentalizing impairments in autism. *Child Development*, *80*(4), 1097–1117. https://doi.org/10.1111/j.1467-8624.2009.01319.x
